# Tau Protein and Neurodegeneration

**DOI:** 10.1002/alz70855_098415

**Published:** 2025-12-23

**Authors:** Michel Goedert

**Affiliations:** ^1^ MRC Laboratory of Molecular Biology, Cambridge, Cambridgeshire, United Kingdom

## Abstract

**Background:**

In 1988, we showed that tau protein is an integral component of the paired helical filaments (PHFs) of Alzheimer's disease. Over thirty neurodegenerative diseases are now known to exhibit filamentous tau inclusions. We identified the six tau isoforms that are produced in the adult human brain from a single gene by alternative mRNA splicing and showed that they are present in PHFs. The relevance of tau became clearer in 1998, when three papers showed that tau gene mutations cause ‘frontotemporal dementia and parkinsonism linked to chromosome 17’ (FTDP‐17). Sixty‐five different mutations have now been identified and filamentous tau inclusions are always present. This work has made it possible to produce transgenic mouse lines that form filamentous tau inclusions and exhibit neurodegeneration. We produced such a line that also led us to demonstrate that filamentous tau exhibits prion‐like properties in the brain.

**Method:**

Since 2017 we are determining the near‐atomic structures of tau filaments from human brains by electron cryo‐microscopy (cryo‐EM).

**Result:**

The different Alzheimer and Pick tau folds establish the existence of molecular conformers of assembled tau. Even though tau filaments from the brains of individuals with chronic traumatic encephalopathy (CTE) are also made of all six brain tau isoforms, the CTE fold is distinct from the Alzheimer fold. Sporadic four‐repeat tauopathies can be divided into those with four‐layered structures (corticobasal degeneration and argyrophilic grain disease) and those with three‐layered structures (progressive supranuclear palsy and globular glial tauopathy). It is also possible to identify new disease entities (limbic‐predominant neuronal inclusion body four‐repeat tauopathy) through their distinct folds.

**Conclusion:**

Each sporadic tauopathy has a specific fold, but several diseases can share a given fold. To study mechanisms of disease, we are attempting to form the human disease folds in the test tube. Known structures of tau filaments assembled *in vitro* and *in vivo* are different from those found in the human brain. So far, we have managed to form the Alzheimer fold following the assembly of truncated or modified full‐length human tau.

